# Portable Multi-Layer Capsule-Shaped Triboelectric Generator for Human Motion Energy Harvesting

**DOI:** 10.3390/mi15070852

**Published:** 2024-06-29

**Authors:** Xinglin Yang, Da Huo, Jianye Su, Zhouyu He

**Affiliations:** School of Energy and Power, Jiangsu University of Science and Technology, Zhenjiang 212003, China; 221110803105@stu.just.edu.cn (D.H.); 221110803108@stu.just.edu.cn (J.S.); 221110803104@stu.just.edu.cn (Z.H.)

**Keywords:** triboelectric generator (TEG), multi-layer capsule structure, human motion energy, portable, modular

## Abstract

This paper introduces a novel portable multi-layer capsule-shaped triboelectric generator (CP-TEG), aimed at optimizing the performance of triboelectric generator technology in terms of miniaturization, modularity, and efficient energy collection. The CP-TEG utilizes a unique multi-layer, stacked structure and an elliptical cylindrical design to increase the effective frictional area and enhance power generation efficiency. Its portable design allows for flexible application in various environments and scenarios. Experimental results demonstrate that the CP-TEG can maintain stable and efficient electrical output under various motion amplitudes and frequencies, and it shows good adaptability to the direction of motion excitation. With a motion amplitude of 7 cm and a frequency of 1.94 Hz, the CP-TEG can charge a 220 μF capacitor to 1.3 V within 100 s. The power generation unit’s output voltage and current are more than three times higher than that of traditional single-layer contact-separation mode triboelectric devices. Particularly, its performance in harvesting energy from human motion underscores its effectiveness as a renewable energy solution for wearable devices. Through its innovative structural design and optimized working mechanism, the CP-TEG demonstrates excellent energy collection efficiency and application potential, offering new options for sustainable energy solutions and development.

## 1. Introduction

With the rapid development of wearable devices, electronics, and IoT technology, the demand for miniaturized, portable, and efficient energy conversion technologies is increasing [1,2,3,4]. The popularity of these technologies not only brings convenience but also poses new demands and challenges, especially in environments where the energy supply is limited. How to effectively harvest energy from the surrounding environment and convert it into electrical energy has become a key issue explored by researchers [5,6,7,8,9,10,11]. Currently, electromagnetic generators, piezoelectric generators, and triboelectric generators (TEGs) can all be used to harvest vibration energy. However, electromagnetic and piezoelectric generators have lower output at lower frequency ranges (below 4 Hz). In contrast, triboelectric generation technology (TEG) has garnered widespread attention due to its lightweight, high efficiency, low cost, and relatively high output in low-frequency ranges [12,13,14,15,16,17,18,19,20].

Triboelectric generator technology uses the triboelectric effect and electrostatic induction principles to convert mechanical energy into electrical energy through friction or contact [9,21]. Since its first introduction in 2012 [22,23,24], researchers have significantly improved the energy conversion efficiency and application scope of TEGs through material innovation [25,26,27,28] and structural design optimization [29,30,31,32,33]. Concurrently, the advancement of this technology in emerging fields has also heightened expectations for new applications in energy harvesting [34].

Despite significant advancements in TEG technology, there remain considerable challenges in achieving efficient energy harvesting to meet the demands of wearable devices and portable electronics [35,36,37]. One such challenge is designing TEG devices that are not only compact but also capable of efficient energy collection, especially in scenarios with limited space or diverse movement patterns [38,39,40]. Jihoon Chung et al. [41] proposed a manual gyroscope hybrid generator for charging portable devices, which can power portable sensors and smartphones through manual rotation. Inspired by gyroscopes, Qian Tang et al. [42] proposed a new type of triboelectric generator that serves as a reliable portable instant power source for personal health monitoring devices, capable of converting low-frequency pulling motions into high-frequency rotations through manual pulling. Yongjiu Zou et al. [43] introduced a portable triboelectric generator utilizing gyroscopic dynamics, which harnesses energy from low-frequency and linear biomechanical movements through gyroscopic rotation. However, these triboelectric generators require manual pulling to provide an additional linear driving force, resulting in no output when carried in a backpack daily. Therefore, designing a triboelectric generator that can harvest complex human movements without the need for additional linear driving forces is crucial as a portable power source for wearable devices. As the excitation frequency of portable devices by human motion is low, the energy harvesting capability of TEGs is limited at low frequencies without components such as springs. By incorporating spring components in TEGs, the potential energy during mechanical triggering can be stored and converted into electrical energy multiple times, thus enhancing the efficiency of energy collection. Furthermore, enhancing the modularity and stability of portable TEG devices, as well as reducing the difficulty of manufacturing TEGs, are key challenges that need to be addressed in current research.

Addressing these issues, this study introduces a novel portable multi-layer, capsule-shaped triboelectric generator that can collect energy from complex human movements, thus enhancing the miniaturization, modularity, and efficiency of TEG technology. This device employs a unique multi-layer design, significantly increasing the effective friction area. The output voltage of the power generation module can reach 250 V, and the output current can reach 7.2 μA. Compared to the traditional single-layer contact-separation mode, the output is more than tripled. The introduction of the central heavy ball can store the potential energy of a single movement and release it multiple times, thus improving power generation efficiency. Additionally, the introduction of conical springs improves the device’s adaptability to the direction of motion stimuli. Its portable capsule casing allows for flexible application in various settings, such as backpacks and handbags, providing a stable power supply for wearable and other small electronic devices.

## 2. Experimental Section

### 2.1. Fabrication of the CP-TEG

The CP-TEG consists of two independently replaceable TEG modules, each comprising four TEG units. Each unit is made up of a circular copper foil electrode, a PTFE film, and a PET film. Foam is introduced under the electrode and adhered to a 3D-printed circular PLA support board. The layers of support boards are connected by three springs. The upper and lower parts of the TEG modules are connected by conical springs and a central steel ball and are fixed to the top and bottom ends of 3D-printed support structures within a capsule ball casing. Supplementary Figure S1 displays the support structures. The capsule is sealed to ensure stable output. The specifications of the CP-TEG are shown in Table 1.

### 2.2. Electrical Measurements of the CP-TEG

The electrical output performance of the CP-TEG was measured using an electrometer (6514, Keithley, Cleveland, OH, USA). A data acquisition card (DAQ-USB3214) and a C#-based software platform (DAQ-Explorer Rev 2.2.0) were used for data collection and analysis. A 3D printer (Kingroon KLP1) was utilized to fabricate support boards and structures. A commercial motor (RS-555 50 W) was used to drive the CP-TEG.

## 3. Results and Discussion

### 3.1. Structure and Working Principle of the CP-TEG

**Figure 1:**
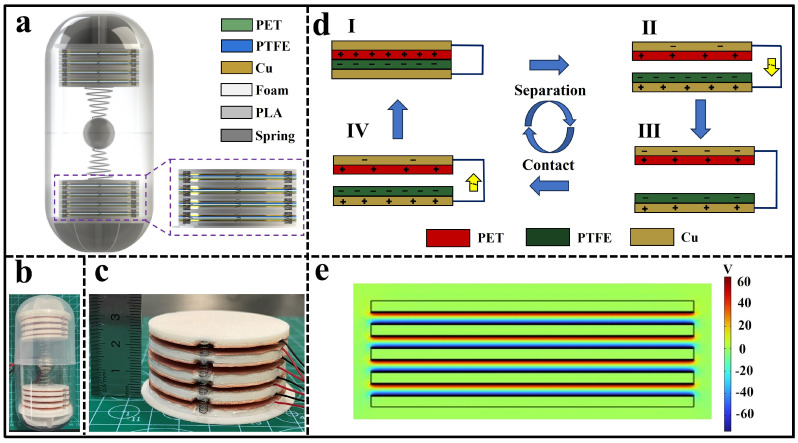
Schematic diagram of the working principle and structure of CP-TEG. (**a**) CP-TEG model. (**b**) Physical model of the CP-TEG. (**c**) Physical model of a module of the CP-TEG. (**d**) Charge distribution in a unit of the CP-TEG during contact-separation motion (State I-II represents the separation phase of the power generation unit, State II-III involves the continued separation until the maximum distance is reached, State III-IV is the process of the power generation units coming into contact, and State I to IV encompasses the progression to full contact between the power generation units). (**e**) The potential distribution of the CP-TEG module was simulated using COMSOL Multiphysics software (COMSOL Multiphysics 6.1).

As shown in Figure 1a,b, due to the elliptical cylindrical shape, which is easy to carry and responsive to movement, the CP-TEG was designed with a multi-layered stacked structure inside, facilitating more efficient energy collection. The CP-TEG contains eight TEG units, divided into two modules, each consisting of four units, as depicted in Figure 1c. Each module is independently replaceable, reducing maintenance costs. Supplementary Figures S2 and S3, respectively, display the physical structure and exploded view models of the TEG modules. The upper and lower modules are connected by conical springs to a central steel ball and are fixed to the support structures, allowing each TEG unit to complete contact-separation motion driven by the central steel ball. A layer of foam is placed beneath the electrode of each TEG unit, enhancing contact between units in practical applications [44]. The introduction of springs between the TEG units helps maintain a stable separation of the contact surfaces of the membranes, and in the absence of motion, keeps the units separated, thus increasing the device’s lifespan.

The working mechanism of the CP-TEG is shown in Figure 1d, where each basic unit consists of a PTFE and PET film, operating in a contact-separation mode triggered by motion. In closed state I, when the PET film contacts the PTFE film, triboelectric charging occurs, with the PTFE surface becoming negatively charged and the PET surface positively charged. During the separation from state II to III, the negative charge on the PTFE surface and the positive charge on the PET surface induce a potential difference between the two copper electrodes, driving the positive charge from the PET side electrode to the PTFE side electrode. When the PET and PTFE films close again in state IV, the potential difference between the two electrodes gradually decreases, and the induced charges return. Each TEG unit operates in motion triggered by different stimuli, causing continuous contact-separation movement of the two surfaces, generating pulsed alternating current. As illustrated in Figure 1e, the potential distribution across the two electrodes was simulated using COMSOL Multiphysics software. The potential distribution diagram clearly shows the voltage difference between the two electrodes, which will drive the current flow in the external circuit. Supplementary Video S1 displays a demonstration video of the CP-TEG module lighting up LEDs.

### 3.2. Performance of the CP-TEG

**Figure 2:**
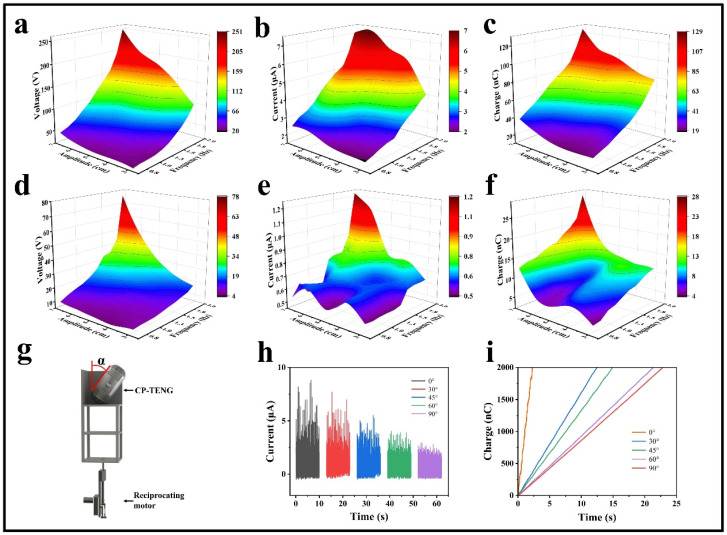
The impact of frequency, excitation amplitude, and excitation angle on the output of the CP-TEG. (**a**–**c**) Output performance of the lower half module of the CP-TEG at frequencies of 0.56 Hz to 1.94 Hz and amplitudes of 3 cm to 7 cm. (**d**–**f**) Output performance of the upper half module of the CP-TEG at frequencies of 0.56 Hz to 1.94 Hz and amplitudes of 3 cm to 7 cm. (**g**) Installation method of the CP-TEG on a frame driven by a variable speed reciprocating motor. (**h**) Output current of the CP-TEG at different α angles. (**i**) Charge accumulation performance of the CP-TEG at different α angles.

To measure the output performance of the CP-TEG, it is mounted on a frame driven by an adjustable speed reciprocating motor. By varying the stroke and frequency of the reciprocating motor, different motions’ effects on the CP-TEG’s output performance are simulated, primarily collecting motion energy perpendicular to the contact-separation surface of the TEG units. The amplitude of the reciprocating motor is set from 3 cm to 7 cm, and the frequency ranges from 0.56 Hz to 1.94 Hz. Measurements were taken separately for the upper and lower modules of the CP-TEG. Figure 2a–c show the output of the four TEG units in the lower module of the CP-TEG, and Figure 2d–f display the output of the upper module, including the relationships of output voltage, current, and transferred charge to the frequency and amplitude of linear motion. The results indicate that when the motion amplitude exceeds 5 cm, the lower module of the CP-TEG has a relatively stable high output within the frequency range of 1.5–1.94 Hz, while the upper module reaches a relatively high and stable output only when the motion amplitude exceeds 6 cm, within the same frequency range. This is due to the gravitational effects of the individual TEG units and the central iron ball, resulting in a smaller average distance between the modules in the lower part of the CP-TEG compared to the upper modules. This allows the units in the lower module to complete the contact-separation movement at smaller motion amplitudes, enabling more thorough contact between the units; thus, the optimal amplitude for the lower modules is less than that for the upper modules. Specifically, the lower module of the CP-TEG, at a motion amplitude of 7 cm and a frequency of 1.94 Hz, can achieve a maximum open-circuit voltage of 250 V, a short-circuit current of 7.2 μA, and a transferred charge of 129 nC. The upper module of the CP-TEG, under the same conditions, can reach an open-circuit voltage of 78 V, a short-circuit current of 1.22 μA, and a transferred charge of 28 nC.

Since CP-TEG cannot always move vertically in practical scenarios, the overall output performance of the device is affected when the direction of motion stimulation forms an angle with the vertical orientation of the CP-TEG. It is crucial to maintain good output performance under various motion stimulations. Compared to origami structures, the stacked structure of the internal power generation modules in CP-TEG enhances its adaptability to the direction of motion stimuli, ensuring that each group of power generation units produces electrical energy with each motion stimulus. To investigate the effect of motion stimulation direction on the overall output performance of the CP-TEG, as shown in Figure 2g, the CP-TEG is mounted on a frame driven by an adjustable speed reciprocating motor. The amplitude and frequency of the motor are kept constant at 7 cm and 1.94 Hz, respectively, while the angle α between the CP-TEG and the vertical direction is varied to simulate different scenarios of motion. As shown in Figure 2h, the angles α are set to 0°, 30°, 45°, 60°, and 90°, where α = 0° represents the vertical position of the CP-TEG, and α = 90° represents the horizontal position. To measure the overall current output of the CP-TEG, the upper and lower modules are rectified and connected in parallel. As the angle α increases, the overall current output of the device gradually decreases. At angles of α = 0°, 30°, 45°, 60°, and 90°, the overall current outputs of the CP-TEG are 8.8 μA, 7.7 μA, 5.5 μA, 4 μA, and 2.9 μA, respectively. The results confirm the CP-TEG device’s good adaptability to the direction of motion stimulation, enhancing its applicability in various scenarios and reducing its dependence on a single type of motion. Figure 2i shows the charge accumulation rate of the CP-TEG device at angles of motion stimulation relative to the vertical at 0°, 30°, 45°, 60°, and 90°. The results indicate that as angle α gradually increases, the charge accumulation rate decreases. When the CP-TEG accumulates a transferred charge of 2000 nC, the time taken at angles α of 0°, 30°, 45°, 60°, and 90° are, respectively, 2.3 s, 12.5 s, 14.9 s, 21.3 s, and 22.9 s. This outcome is expected as, with increasing angle α, the contact area between TEG units under the same motion stimulation decreases, resulting in insufficient contact and reduced transferred charge.

**Figure 3:**
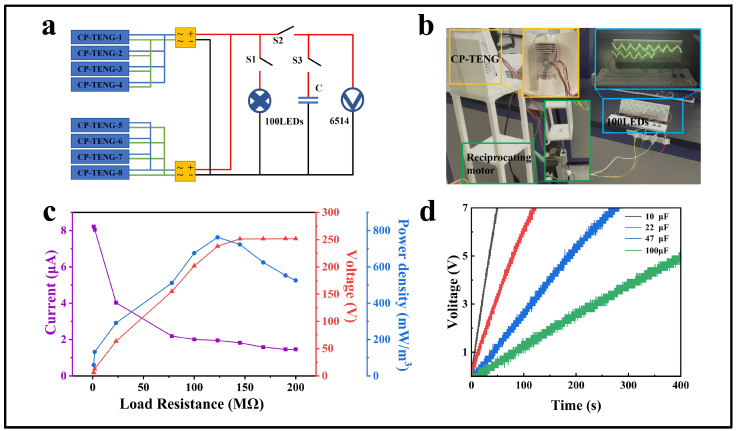
Output characteristics and demonstration of the motor-driven CP-TEG. (**a**) Schematic diagram of the circuit connections of the CP-TEG. (**b**) Photo of the CP-TEG powering 100 LEDs. (**c**) Output voltage, output current, and peak power of the CP-TEG with resistance driven by a linear motor. (**d**) Comparison of charging times for capacitors of different capacities by the CP-TEG.

The circuit connection of the CP-TEG during practical application is shown in Figure 3a. In both the upper and lower modules of the CP-TEG, each unit is connected in parallel and then rectified to convert AC to DC before connecting to electrical appliances. Since the operational phases of each working unit in the modules are the same, the units can be connected in parallel before rectification. As shown in Figure 3a, under the drive of the reciprocating motor with an amplitude of 7 cm and frequency of 1.94 Hz, when switch S1 is closed and S2 is open, the CP-TEG successfully lights up 100 LEDs, as shown in Figure 3b and Supplementary Video S2. As shown in Figure 3d, when the CP-TEG is used to charge capacitors, under the drive of the reciprocating motor with an amplitude of 7 cm and a frequency of 1.94 Hz, by turning on switch S1 and closing switches S2 and S3, the CP-TEG demonstrates good charging capabilities. A small capacitor of 10 μF can be charged to 7 V within 50 s. For a 220 μF capacitor, the voltage reaches 1.3 V after charging for 100 s. The charging performance of triboelectric devices is also an important indicator of their capability. As shown in Supplementary Figure S8, comparing the charging performance of CP-TEG with several other triboelectric devices that collect kinetic energy demonstrates a significant improvement in the charging performance of CP-TEG for capacitors.

Additionally, the dependence of the output peak power of the CP-TEG on the external load resistance was studied. Figure 3c illustrates the trends in output voltage, output current, and peak power density of a single CP-TEG with varying external load resistances. As the load resistance increased from 1 Ω to 200 MΩ, the output voltage gradually increased, and the peak power output initially increased and then decreased. When the external load resistance of CP-TEG is 1 MΩ, the peak power density reaches a maximum of 59.5 mW/m^3^, with a corresponding voltage output of 6 V and an output current of 8.2 μA. The maximum peak power density of CP-TEG is 763 mW/m^3^, with a corresponding voltage output of 237 V and an output current of 2 μA, with a matched resistance of 120 MΩ. At this condition, the average output voltage of CP-TEG is 84 V, and the average output current is 0.44 μA. Additionally, the power under cyclic input vibrations of the CP-TEG device is also an important criterion for evaluating its electrical energy output. Therefore, we studied the dependency of the CP-TEG’s average power on external load resistance, as shown in Supplementary Figure S6. As the load resistance gradually increases, the average output power of the CP-TEG also increases, reaching a maximum of 0.058 mW at a resistance value of 120 MΩ. As the load resistance continues to increase, the average output power of the CP-TEG gradually decreases, demonstrating the impedance matching and output performance of the CP-TEG under cyclic input vibrations. Supplementary Figures S4 and S5 display the average output voltage and current of CP-TEG under different load conditions. The output performance under motion excitation at different frequencies and different matching impedances is shown in Supplementary Figure S7. The power density of the CP-TEG increases with the increase in excitation frequency. It is noteworthy that the optimal matching impedance remains 120 MΩ at different frequencies.

**Figure 4:**
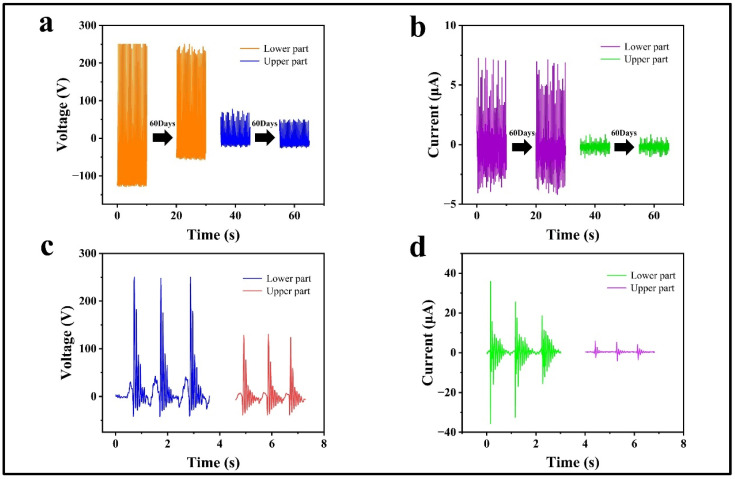
Output characteristics of the CP-TEG under special conditions. (**a**,**b**) Performance comparison of the upper and lower modules of the CP-TEG after being left for 60 days. (**c**,**d**) Output characteristics of the upper and lower modules of the CP-TEG during impact motion.

Given the intermittent usage of CP-TEG in daily applications, to study the stability of the CP-TEG device’s output, as shown in Figure 4a,b, measurements were made on the same CP-TEG after normal use and 60 days of inactivity. The results show that the peak output voltage of the module beneath the CP-TEG decreased from 250 V to 244 V, a reduction of 2.4%, and the peak current output decreased from 7.2 μA to 7.1 μA, a reduction of 1.4%. The peak output voltage of the module above the CP-TEG decreased from 78 V to 51 V, a reduction of 35%, and the peak current output decreased from 1.1 μA to 1 μA, a reduction of 9%. The above results confirm the durability of the CP-TEG device under intermittent use conditions and also demonstrate good long-term performance reproducibility. Due to potential impacts or collisions in specific scenarios, the impact resistance of the CP-TEG device was investigated. As depicted in Figure 4c,d, three-spaced impact tests were conducted on the CP-TEG, measuring the output performance of both the upper and lower modules separately. The results showed that in response to impact movements, the CP-TEG device exhibited superior output performance; the lower module achieved an output voltage of 250 V and a current of 35 μA, while the upper module reached 130 V and 5 μA, showing overall higher performance compared to regular motion stimuli. Following a single impact, the springs between each module of the CP-TEG caused the output voltage and current waveforms to exhibit multiple gradually decreasing peaks, resulting in multiple reductions in output, thereby enhancing the CP-TEG’s output capacity.

### 3.3. Portable Applications of CP-TEG

**Figure 5:**
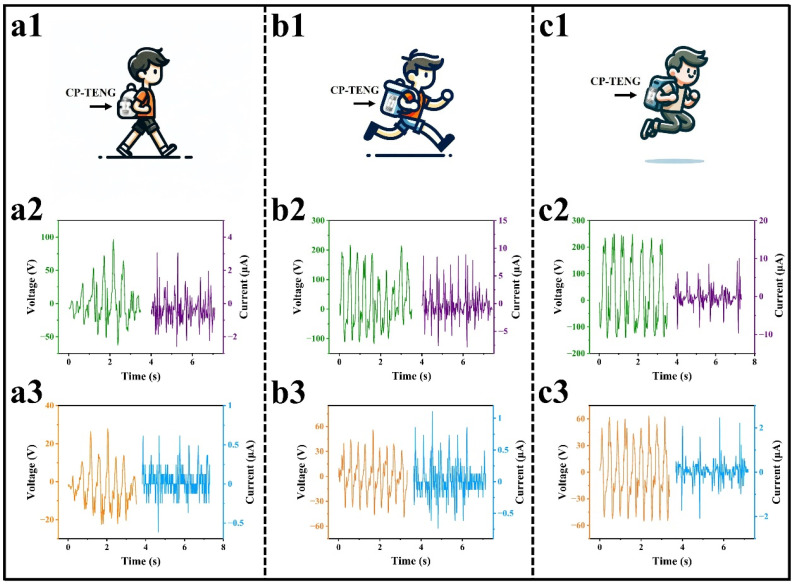
CP-TEG harvests human motion energy. (**a1**,**b1**,**c1**) Each shows the motion modes used to excite the CP-TEG. (**a2**,**b2**,**c2**) Each shows the output performance of the lower half module of the CP-TEG in different motion modes. (**a3**,**b3**,**c3**) Each shows the output performance of the upper half module of the CP-TEG in different motion modes.

The multi-layer capsule-type triboelectric generator (CP-TEG) features a lightweight, low-cost, simple structure, and is small in size, making it suitable as a portable energy generation device for powering wearable human sensor devices. Figure 5 illustrates the application of biomechanical energy harvesting. Figure 5a1,b1,c1 show the CP-TEG placed in a backpack net, used to collect energy from various human movements, including walking, running, and jumping. It is evident that the CP-TEG generates electrical output regardless of the type and intensity of the movement. Supplementary Figure S9 presents snapshots of the demonstration of biomechanical energy harvesting connected to multiple LEDs in series. Figure 5a2,a3,b2,b3,c2,c3 display the output voltage and current from the upper and lower modules of the CP-TEG under various motion modes. During walking, the voltage outputs were 28 V and 96 V with currents of 0.6 μA and 3 μA, respectively; during running, 56 V and 217 V with currents of 1.1 μA and 8.8 μA; and during jumping, 63 V and 248 V with currents of 2.5 μA and 10 μA. These figures demonstrate the output capability of the CP-TEG in various motion modes, providing a continuous supply of renewable energy for wearable smart devices.

## 4. Conclusions

In conclusion, we have designed and fabricated a capsule-shaped TEG with a multi-layer stacked structure, which can harvest energy from various human motions without the need for additional linear power input and demonstrates good performance. The multi-layer stacked structure increases the space utilization and power density of the TEG. The springs between the power generation units provide excellent elasticity and self-rebound performance, making the entire device compact and lightweight. Compared to traditional contact-separation triboelectric generators, it has significantly improved performance output. The modular design of the two TEGs, placed on the top and bottom, facilitates device maintenance and internal module replacement. The central conical spring and steel ball ensure the device’s sensitivity to motion stimuli, while also enhancing the contact area between the two membranes. Comprehensive studies were conducted on the designed CP-TEG for continuous simulated motion stimuli, special scenarios of impact or tapping motion stimuli, and its application in harvesting energy from human movements. For motion with fixed amplitude and frequency simulated by a reciprocating motor, a single CP-TEG module can generate a maximum open-circuit voltage of 250 V, a short-circuit current of 7.2 μA, and a transferred charge of 129 nC. With an external matching resistance of 120 MΩ, the CP-TEG achieves a peak power density of 763 mW/m^3^, with a corresponding voltage output of 237 V. Owing to its lightweight, long-term durability, and excellent portability, the proposed CP-TEG has successfully demonstrated its application in harvesting energy from human movements. As research on further miniaturization of CP-TEG progresses, it is expected to find applications in fields such as biomedical engineering.

## Figures and Tables

**Table 1:** CP-TEG device specifications.

Name	Specification
Capsule Shell	Outer diameter: 70 mm, Height: 147 mm
Copper Foil Electrode	Thickness: 60 μm, Radius: 30 mm
PTFE Film	Thickness: 50 μm, Radius: 30 mm
PET Film	Thickness: 50 μm, Radius: 30 mm
Foam	Thickness: 1 mm, Radius: 30 mm
PLA Support Plate	Thickness: 1 mm, Radius: 30 mm
Spring	Length: 5 mm, Width: 2 mm, Wire diameter: 0.02 mm
Steel Ball	Diameter: 22 mm, Weight: 40.5 g
Conical Spring	Small outer diameter: 8 mm, Large outer diameter: 16 mm, Length: 20 mm
Lateral Support Structures	Diameter: 62 mm, Height: 21 mm
Maximum Charge Accumulation Rate	0.87 μC/s
Capacitor Charging Speed	390 s to charge a 100μF capacitor to 5 V
Module Peak Voltage	250 V
Module Peak Current	7.2 μA
Optimal Matching Resistance	120 MΩ
Peak Power Density	763 mW/m^3^
Optimal Operating Frequency Range	Greater than 1.5 Hz

## Data Availability

Data are contained within the article and Supplementary Materials.

## Supplementary Materials

The following supporting information can be downloaded at: https://www.mdpi.com/article/10.3390/mi15070852/s1, Figure S1: support structures; Figure S2: physical structure diagram of CP-TEG module; Figure S3: exploded view diagram of CP-TEG module; Figure S4: average output voltage of CP-TEG under different load conditions; Figure S5: average output current of CP-TEG under different load conditions; Figure S6: average output power of the CP-TEG under different loads; Figure S7: the output performance under motion excitation at different frequencies and different matching impedances; Figure S8: comparison of the charging performance of several triboelectric energy harvesting devices for a 10μF capacitor over 50 s; Figure S9: snapshots of a demonstration where a CP-TEG collects biomechanical energy to illuminate LEDs; Video S1: demonstration video of CP-TEG module lighting up LEDs; Video S2: the CP-TEG successfully lights up 100 LEDs.
