# Supplementary material for: Portable Multi-Layer Capsule-Shaped Triboelectric Generator for Human Motion Energy Harvesting
Source: Micromachines (Basel). 2024 Jun 29;15(7):852. doi: 10.3390/mi15070852 (PMC11278884; doi:10.3390/mi15070852)
Supplement: Supplementary file 1 [file micromachines-15-00852-s001.zip › micromachines-3052632 supplementary.pdf]

micromachines

MDPI

Supplementary Information: Portable Multi-Layer Capsule-Shaped Triboelectric Generator for Human Motion Energy Harvesting

Xinglin Yang *, Da Huo, Jianye Su and Zhouyu He

School of Energy and Power, Jiangsu University of Science and Technology, Zhenjiang 212003, China; 221110803105@stu.just.edu.cn (D.H.); 221110803108@stu.just.edu.cn (J.S.); 221110803104@stu.just.edu.cn (Z.H.)

* Correspondence: yangxl23320@163.com; Tel.: +86-1890-5280-992

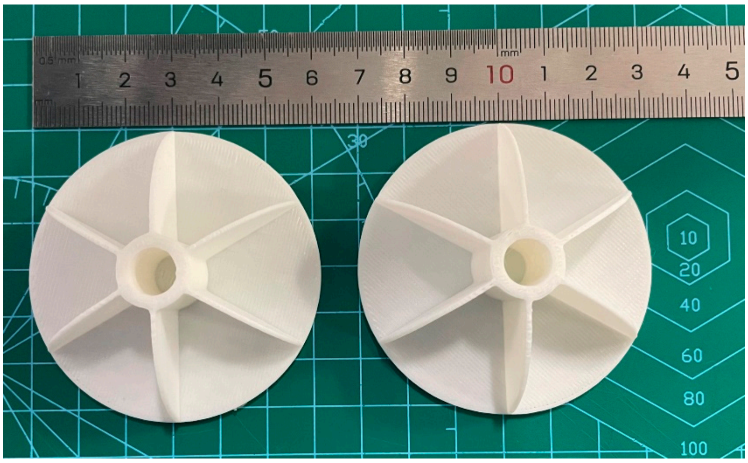

Figure S1. Support structures.

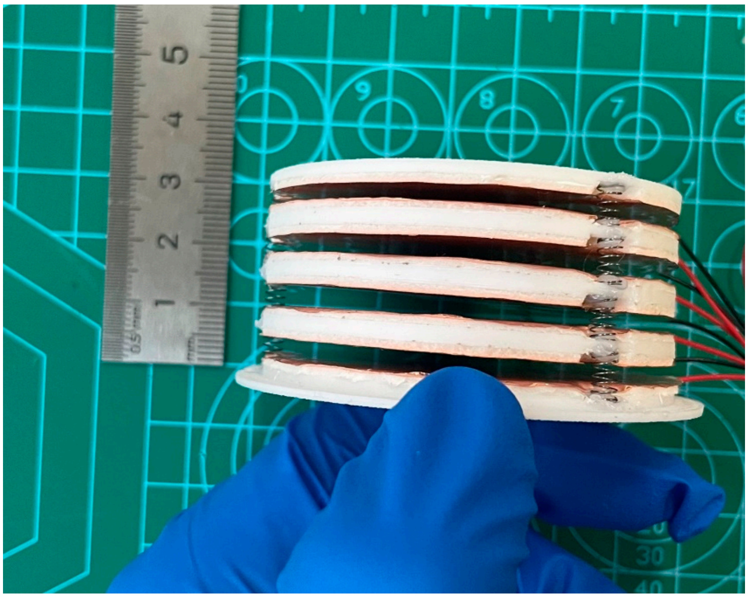

Figure S2. Physical structure diagram of CP-TEG module.

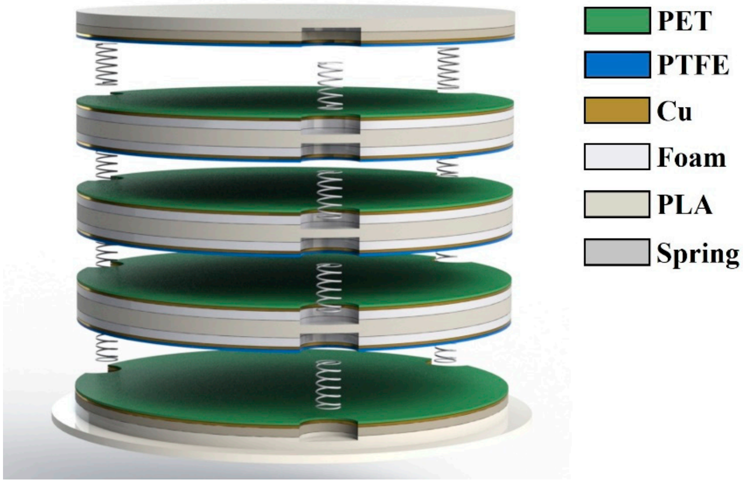

Figure S3. Exploded view diagram of CP-TEG module.

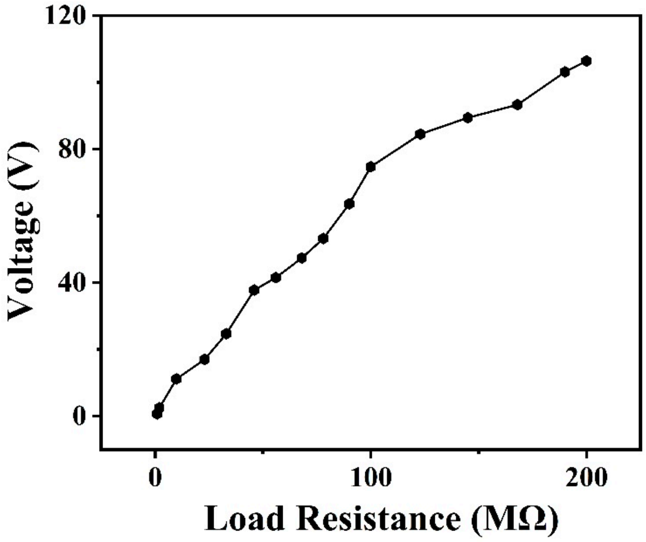

Figure S4. Average output voltage of CP-TEG under different load conditions.

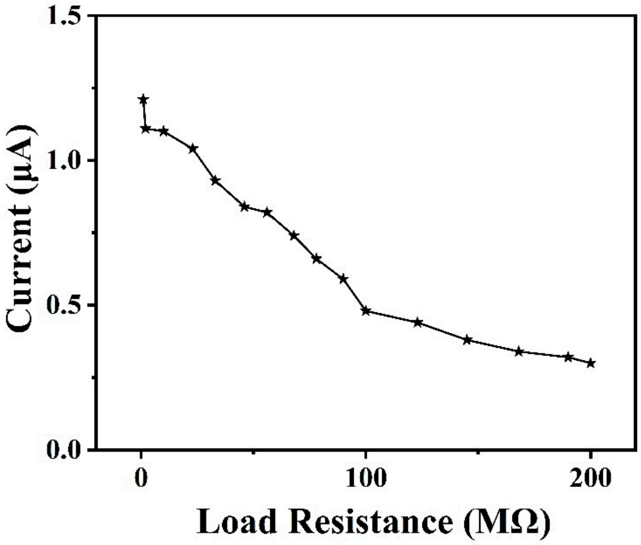

Figure S5. Average output current of CP-TEG under different load conditions.

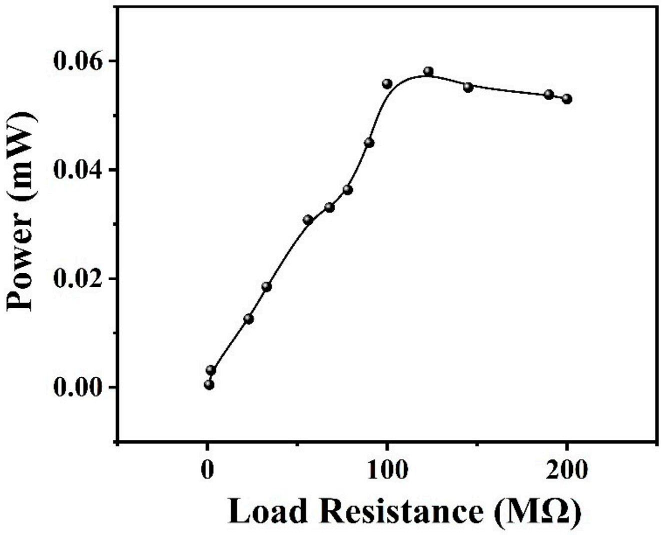

Figure S6. Average output power of the CP-TEG under different loads.

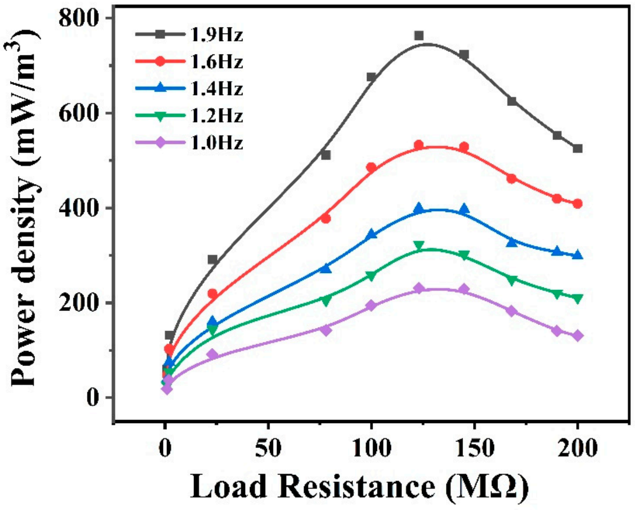

Figure S7. The output performance under motion excitation at different frequencies and different matching impedances.

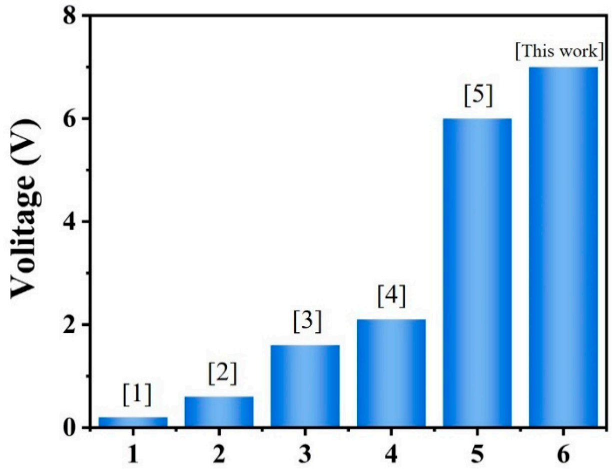

Figure S8. Comparison of the charging performance of several triboelectric energy harvesting devices for a 10μF capacitor over 50 seconds.

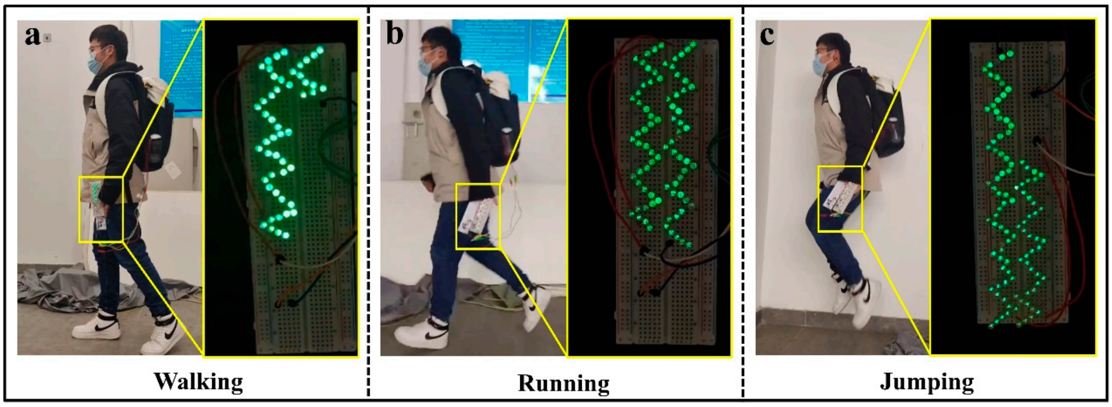

Figure S9. Snapshots of a demonstration where a CP-TEG collects biomechanical energy to illuminate LEDs. (a) In the walking mode of operation, the CP-TEG can illuminate 50 LEDs. (b) In the running mode of operation, the CP-TEG can illuminate 80 LEDs. (c) In the jumping mode of operation, the CP-TEG can illuminate 100 LEDs.

Supplementary Video S1.
Supplementary Video S2.
